# Examining the Relationship Between Obesity, Clinical Tests, and Workplace Productivity in Canadian Long-Haul Truck Drivers

**DOI:** 10.1177/21650799261454291

**Published:** 2026-06-24

**Authors:** Mackenzie L. McKeown, Alexander M. Crizzle

**Affiliations:** 1 School of Public Health, University of Saskatchewan

**Keywords:** long-haul truck driver, workplace productivity, obesity, cognition, clinical assessment

## Abstract

**Background:**

The Canadian trucking industry employs over 300,000 long-haul truck drivers (LHTD) and has the highest number of worker compensation claims of any industry in Canada. Although studies have shown associations between obesity, decreased workplace productivity, and cognitive impairment in the general population, there is limited information on these relationships among LHTD. This pilot study aimed to: (1) profile LHTD health, demographics, and clinical test scores; (2) examine associations between obesity and workplace productivity; and (3) explore connections between obesity and clinical assessment test scores.

**Methods:**

LHTD were recruited from various provincial and federal trucking associations and companies across Canada. In total, 36 LHTD completed a demographic questionnaire that surveyed their work, driving history, and health, followed by a few objective health measures (e.g., height; weight; blood pressure), as well as a battery of cognitive (e.g., Trail Making Test A and B; Montreal Cognitive Assessment; Useful Field of View; Clock Draw Test), visual (e.g., visual acuity; contrast sensitivity), and motor (e.g., Rapid Pace Walk) tests.

**Results:**

Over half of the sample (63.9%) were obese (BMI greater than 30.0 kg/m^2^). Obese LHTD took significantly more sick days (*p* = .022), worked significantly more while sick and injured (*p* = .006 and *p* = .039, respectively), had significantly more crashes (*p* = .014), and exhibited poorer divided attention/concentration (*p* = .014), compared to non-obese LHTD.

**Conclusions/Applications to Practice:**

The findings show that obesity can impact worker productivity in LHTD, but the complex interplay between cognition, obesity, and worker productivity warrants further investigation.

## Introduction

The Canadian trucking industry, which employs over 300,000 long-haul truck drivers (LHTD), is critical to the economy (Gill & Macdonald, 2013; Krasniuk et al., 2024; McKeown & Crizzle, 2025). Unlike other regulated industries (e.g., accommodation and food services; retail), LHTD are subjected to prolonged periods away from home, long work hours (up to 14 hours a day), poor diet and nutrition, and extended periods of sedentary behavior (A. M. Crizzle et al., 2020; Krasniuk et al., 2024; Lemke, Houghtaling, et al., 2023; McKeown & Crizzle, 2025), which are endemic to the occupation (A. Crizzle et al., 2018; Hege et al., 2016). Together, these risk factors impact LHTD health (Bigelow et al., 2014), with prior Canadian and US studies reporting that 32% to 83% of LHTD are obese (body mass index [BMI] of 30 kg/m^2^ or more; A. M. Crizzle et al., 2020, 2024; Hege et al., 2016; Lemke et al., 2015; Ronna et al., 2016; Shaw et al., 2023).

In the general adult population, studies show that obesity is associated with impairments in memory (e.g., episodic and working), learning, executive function (i.e., problem solving and planning), and attention (Dye et al., 2017; O’Brien et al., 2017; Spyridaki et al., 2016). Moreover, studies show that working adults (mean age = 45.2) with cognitive impairment (i.e., executive function, memory, attention, and information processing speed) were significantly more likely to be absent from work or attended work when not healthy resulting in an increased likelihood of mistakes and/or workplace accidents (Chokka et al., 2019; Evans et al., 2014; Lohaus & Habermann, 2019; McIntyre et al., 2015). These cognitive domains, which are essential for LHTD to perform their job safely and efficiently, are assessed using a variety of cognitive tests (e.g., Trail Making Test A and B; Montreal Cognitive Assessment; Clock Draw Test; Useful Field of View; Chokka et al., 2019; Evans et al., 2014; Lohaus & Habermann, 2019; McIntyre et al., 2015). Previous studies show that obese participants (BMI of 30 or greater), compared to healthy weight controls, performed significantly worse on the Montreal Cognitive Assessment (MoCA), a measure of global cognition, with an average MoCA score of 25.1 ± 0.3 (i.e., less than 26 is indicative of mild cognitive impairment; Fan et al., 2019; Lentoor, 2022). Other studies show that compared to healthy weight controls, obese adults performed poorer on the Clock Draw Test (CDT; measures executive function) (Lentoor, 2022), the Trails Making Test A (TMTA; measures visual processing and attention) and B (TMTB; measures divided attention and executive skills) tests (Fergenbaum et al., 2009; Gunstad et al., 2010; Kokkinakis et al., 2021). To date, only one study has investigated the cognitive function of LHTD using the TMTA, TMTB, MoCA, and UFOV (Bhattacharya et al., 2023). LHTD were on average 53 ± 13 years old, obese (mean BMI of 32 ± 6 kg/m^2^), male (90%; Bhattacharya et al., 2023), however, cognitive test performance was never segregated by obesity-status nor was it examined with work productivity.

Within the general working population, prior studies report that obese workers are more likely to be absent from work (Borak, 2011; Lehnert et al., 2013; Neovius et al., 2009; Sullivan et al., 2008; van Duijvenbode et al., 2009) and are less productive at work (i.e., presenteeism; Borak, 2011; Shrestha et al., 2016; Sullivan et al., 2007). Workplace productivity consists of both absenteeism (i.e., missed work days due to illness or injury) and presenteeism (i.e., attending work while sick or injured; Borak, 2011), with the Canadian trucking industry reporting the highest number of worker compensation claims, compared to all other industries (Employment and Social Development Canada, 2024), with 11.4 days lost per worker, per year, due to illness or injury (Statistics Canada, 2022). Additionally, prior studies show that obese workers possess more mobility limitations (e.g., reduced range of motion; difficulty with lower limb movements; Backholer et al., 2012) with obese individuals performing significantly worse on the Rapid Paced Walk Test (RPWT; i.e., slower than the cut-point of 7 seconds) compared to their non-obese counterparts (Pataky et al., 2014).

Although prior studies show an association between BMI, cognition, and decreased workplace productivity in the general adult population, studies have yet to examine whether cognition is related to obesity-status (i.e., obese vs. non-obese) or with poorer worker productivity in LHTD. Based on the prior studies that show an association between obesity and work productivity in the general population (Borak, 2011; Cournot et al., 2006; Dye et al., 2017; Lehnert et al., 2013; Neovius et al., 2009; O’Brien et al., 2017; Pataky et al., 2014; Shrestha et al., 2016; Spyridaki et al., 2016; Sullivan et al., 2007, 2008; van Duijvenbode et al., 2009; Wang et al., 2016), it is hypothesized that obese LHTD will also have poorer cognition and workplace productivity. Therefore, the objectives of this study are to: (1) profile LHTD health (i.e., obesity and related comorbidities), demographics, and clinical test scores; (2) examine the associations between obesity and workplace productivity (i.e., presenteeism and absenteeism) including sick days; and (3) explore connections between obesity and clinical test scores.

## Methods

### Study Protocol

The Behavioural Research Ethics Board at the University of Saskatchewan” approved the study [REB#2918], which involved LHTD attending a single in-person session that lasted between 60 and 90 minutes (McKeown et al., 2026). Data collection took place from January 2023 to July 2024 at the Driving Research and Simulation Laboratory. Prior to attending the in-person session, LHTD were screened over the phone to verify they were eligible to participate. LHTD were eligible to participate if they: (1) were a permanent resident of Canada or a Canadian citizen; (2) possessed a valid Class 1 or Class A license (i.e., a valid long-haul truck driver license); (3) spent, on average, more than four nights away from home per week due to work; (4) had been working as a LHTD for at least 1 year at the time of the study; and (5) were proficient in English. In total, 36 LHTD participated in the study and completed all aspects of the session.

Once eligible participants arrived for their session, informed consent was obtained and participants first completed a questionnaire collecting information on demographics, work and driving history, and their health, followed by a few objective health measures (e.g., height; weight) assessing their adiposity and blood pressure, as well as a battery of cognitive, visual, and motor tests. Throughout the session, non-caffeinated refreshments (water; zero-sugar ginger ale) were available, and after the session was completed, the participant received a $50 CAD cash honorarium, and their parking fees and taxi fees were covered when applicable.

### Participant Recruitment

Participant recruitment involved the following stakeholders, who either posted the recruitment poster on their social media (e.g., Facebook; X) accounts and/or emailed the poster to their list-serve members/employees: the Saskatchewan Trucking Association, the Alberta Motor Transport Administration, the Manitoba Trucking Association, the British Columbia Trucking Association, Safety Driven British Columbia, the Private Motor Truck Council of Canada, and several Canadian trucking companies. Recruitment posters were distributed to various truck stops around Saskatchewan, and announcements were made weekly on social media platforms (e.g., Facebook; X) and at various trucking events in Canada. Interested participants contacted the research team via email or office phone to undergo screening and enrollment.

### Questionnaire

The questionnaire collected information on participant demographics (e.g., age, ethnicity, province of residence, completed education), fatigue (e.g., sleep quality, minimum and maximum number of hours of sleep in a 24-hour period), and diagnosed medical conditions (e.g., type 2 diabetes, hypertension, depression, and obstructive sleep apnea). Additionally, the questionnaire captured information on the participants’ work and driving history (e.g., years worked as a LHTD, daily working hours, number of days worked per week, total number of driving citations received, crash involvement, and if they were found at-fault for the crash).

### Objective Health Assessments

The objective health measures collected during the session included the participants’ height, weight, body fat percentage, neck circumference, and systolic and diastolic blood pressures. Height and weight were measured using the Seca 700 physician scale and Seca 220 telescopic measuring rod, respectively. Obesity was categorized using the following body mass index (BMI) classifications, calculated from height and weight measures: <18.5 kg/m^2^ was underweight; between 18.5 and 24.9 kg/m^2^ was considered healthy; between 25.0 and 29.9 kg/m^2^ was overweight; and >30 kg/m^2^ was obese (Aswathappa et al., 2013). Obesity was further classified into: (1) Class 1 obesity (BMI range of 30.0–34.9), (2) Class 2 obesity (BMI range of 35.0–39.9), and (3) Class 3 obesity (BMI range of 40.0 or greater; Aswathappa et al., 2013).

Additionally, body fat percentage was obtained using the RENPHO Elis 1 Smart Body Scale and categorized as either low (<10% for males; <20% for females), normal (10%–19.99% for males; 20%–29.99% for females), slightly high (20%–24.99% for males; 30%–34.99% for females), and high (≥25% for males; ≥35% for females; Omron Healthcare Co., Ltd., 2010). Neck circumference was measured once using a tailor’s measuring tape. While standing (with good/proper posture), the participant was instructed to breathe while the measurement was taken. For male participants, the neck circumference measurement was taken just below the laryngeal prominence. For female participants, the measurement was taken midway down the neck. Upper body adiposity was defined as a neck circumference ≥43 cm for men and ≥40 cm for women (Mihaicuta et al., 2021). Lastly, systolic and diastolic blood pressures were each measured twice using the BIOS Ultra Blood Pressure Monitor and then were averaged. Systolic blood pressure categories included normotensive (<135 mmHg) or hypertensive (≥135 mmHg; Rabi et al., 2020). Similarly for diastolic blood pressure, scores were categorized as either normotensive (<85 mmHg) or hypertensive (≥85 mmHg; Rabi et al., 2020).

### Cognitive Tests

The Montreal Cognitive Assessment (MoCA) consists of 11 subtests assessing attention, executive function, memory, language, visuospatial skills, and concentration (Nasreddine, 2022). The MoCA is scored out of 30; higher scores indicate better cognitive functioning (Kokkinakis et al., 2021; Nasreddine, 2022). A cut-point of 26 was used differentiate between normal (≥26) and impaired (<26) cognitive function (Nasreddine, 2022). If a participant had 12 years or fewer of formal education, an additional point was added to their total score; 15 participants had an additional point added to the total MoCA score.

The Trail Making Test A (TMTA) measures visual processing and attention, while the Trail Making Test B (TMTB) measures divided attention and executive skills (Reitan, 1958). Both the TMTA and TMTB are pencil-based tests. TMTA requires the participant to connect numbers in sequential order (e.g., 1–2; 2–3) as fast as they can without lifting their pencil off the paper, whereas the TMTB requires participants to connect both numbers and letters in sequential order (e.g., 1-A-2-B-3-C) as fast as they can (Reitan, 1958). A cut-point of 90 and 180 seconds was used for the TMTA and TMTB, respectively, to differentiate between normal and impaired cognitive function (Bédard et al., 2016; Kokkinakis et al., 2021; Reitan, 1958).

The Clock Draw Test (CDT) is a paper and pencil based test assessing working memory, visuoconstructive ability, and executive function (Hazan et al., 2018; Spenciere et al., 2017). The test requires the participant to draw a clock including the numbers, hands, and appropriate time (e.g., 10 past 11; Freund et al., 2005). The test is scored from 0 to 7; higher scores are better, with a score of less than 5 (i.e., the cut-point) indicating cognitive impairment (Freund et al., 2005).

The Useful Field of View (UFOV) evaluates visual search/processing speed, divided, and selective attention. The UFOV test is administered and scored using a computer and consists of three subtests which increase in difficulty (Posit Science, 2011). The first subtest (UFOV-1) requires the participant to detect and discern a target located centrally that is momentarily shown. During the second subtest (UFOV-2), the participant must identify a target, while also locating a separate target within the periphery. The final subtest (UFOV-3) is a reiteration of UFOV-2; however, the peripheral target is surrounded by distractions. UFOV-1 evaluates visual search and processing speed and has four groupings: (1) 0–30 ms, (2) 31–60 ms, (3) 61–349 ms, and (4) 350–500 ms. A score of 0 to 30 ms represents normal central vision and processing speed; every score after the first grouping indicates slower search and visual processing speed. UFOV-2 evaluates divided attention and has three groupings: (1) 0–99 ms, (2) 100–349 ms, and (3) 350–500 ms. A score of 0–99 ms constitutes normal divided attention ability, with every score after indicating difficulties with divided attention. UFOV-3 assesses selective attention and is scored in three groupings: (1) 0–349 ms; (2) 350–499 ms; and (3) 500 ms. A score that falls within 0–349 ms represents normal selective attention ability, with any score above indicating difficulties with selective attention. Based on the participants’ performance across all three subtests, the program calculates a UFOV Risk Index (RI) based on the developers pre-determined algorithm. This RI assesses the likelihood that a person is unsafe to drive, and is classified into five categories: (1) very low; (2) low; (3) low to moderate; (4) moderate to high; and (5) high (Posit Science, 2011).

### Motor Assessment

The Rapid Paced Walk Test (RPWT) was used to assess participants’ lower limb strength and mobility. The RPWT required participants to walk 10 feet away and back as quickly as possible. Tape on the ground was used to denote the 10-foot distance between the starting and end points. If the participant used a cane or walker, they were instructed to use it if they felt more comfortable, although no participants used any walking aids. The time it took for the participant to complete the test was measured using a stopwatch. A cut-point of 7 seconds was used, with scores greater than 7 seconds indicating poor lower-limb mobility (Pataky et al., 2014).

### Data Analysis

Data were analyzed using the Statistical Package for the Social Sciences (SPSS, Version 28; IBM Corp., 2021). Continuous variables were presented using mean and standard deviation (mean ± *SD*) and range. Categorical variables were presented using frequencies and percentages. Between-group differences in LHTD participants who were obese and not obese were investigated through independent sample’s *t*-tests (if parametric assumptions were met) or Mann-Whitney *U* tests (if parametric assumptions were not met) for continuous variables. For categorical variables, associations were examined through Chi-Square or Fisher’s Exact tests between obese versus non-obese status. Additionally, two-way ANOVA were used to test whether BMI and worker productivity have a significant effect on clinical test scores. All results were presented with *p*-values of ≤.05 to indicate statistical significance.

## Results

### Sample Description

As seen in Table 1, the mean age of the sample was 47.9 ± 12.3 years (range 22–69); 94.4% were men and 77.8% were Caucasian. Participants worked on average 16.6 ± 12.1 years (range 2–48) as a LHTD, working 14.0 ± 1.6 hours (range 9–17) per day, and 6.0 ± 0.9 days (range 4–7) per week. Additionally, participants received 3.4 ± 4.7 (range 0–20) driving citations within their career or 0.2 ± 0.3 (range 0–1.5) driving citations per year of work experience, with over half of the sample (52.8%) having been involved in a crash. Of those involved in a crash, 52.6% were found at-fault.

**Table 1.** Sample Description

Variable	*N* = 36
Age	47.9 ± 12.3 (22.0–69.0)
Gender	
Male	34 (94.4%)
Female	2 (5.6%)
Ethnicity	
Caucasian	28 (77.8%)
Métis	5 (13.9%)
First Nations	2 (5.6%)
Latin American	1 (2.8%)
Province of residence	
Saskatchewan	27 (75.0%)
Alberta	3 (8.3%)
Manitoba	2 (5.6%)
Ontario	2 (5.6%)
British Columbia	2 (5.6%)
Level of education	
Some high school	6 (16.7%)
High school	9 (25.0%)
Some technical/vocational training	6 (16.7%)
Diploma/certificate	3 (8.3%)
Some university	8 (22.2%)
Bachelor’s degree	4 (11.1%)
Work experience	
Years worked as a LHTD	16.6 ± 12.1 (2.0–48.0)
Daily working hours	14.0 ± 1.6 (9.0–17.0)
Days worked per week	6.0 ± 0.9 (4.0–7.0)
Number of driving citations received	3.4 ± 4.7 (0.0–20.0)
Been involved in a crash	19 (52.8%)
Diagnosed medical conditions	
Hypertension	9 (25.0%)
Sleep apnea	5 (13.9%)
Type 2 diabetes	2 (5.6%)
Depression	9 (25.0%)
Body mass index (BMI)	32.2 ± 5.7 (21.0–49.2)
Healthy	1 (2.8%)
Overweight	12 (33.3%)
Class 1 Obese	13 (36.1%)
Class 2 Obese	6 (16.7%)
Class 3 Obese	4 (11.1%)
Neck circumference (cm)	41.4 ± 4.4 (29.0–51.0)
Body fat percentage (%)	31.4 ± 9.5 (13.1–57.0)
Systolic blood pressure (mmHg)	136.1 ± 16.6 (108.5–174.5)
Normotensive	21 (58.3%)
Hypertensive	15 (41.7%)
Diastolic blood pressure (mmHg)	85.5 ± 12.4 (66.5–116.5)
Normotensive	18 (50.0%)
Hypertensive	18 (50.0%)

*Note.* LHTD = long-haul truck drivers. Continuous variables are shown as mean ± *SD* (range). Categorical variables are shown as frequencies (%).

Overall, 63.9% of the sample were classified as being obese (i.e., BMI greater than 30.0) and 36.1% were classified with upper body adiposity (neck circumference ≥43 cm in males and ≥40 cm in females). In total, 30.6% of the sample (i.e., 11 LHTD) had both a BMI greater than 30.0 and a neck circumference greater than 43 cm. LHTD categorized as obese had significantly larger mean neck circumference (*p* = .022) compared to non-obese LHTD (42.6 cm vs. 39.1 cm). Only 25.0% of the sample reported being diagnosed with hypertension by a medical doctor or healthcare professional with one third (33.3%) of those diagnosed with hypertension taking anti-hypertensive medication (e.g., amlodipine; felodipine; nifedipine) to control their blood pressure. Based on objective measures, 41.7% and 50.0% of participants had hypertensive systolic and diastolic blood pressures, respectively. Only 5.6% of the sample were diagnosed with Type 2 diabetes and 13.9% were diagnosed with OSA; 60% used a continuous positive airway pressure (CPAP) machine while sleeping.

### Clinical Test Scores

All clinical test scores are shown in Table 2. The sample had adequate visual acuity and contrast sensitivity based on the licensing standards in Canada. On the RPWT, four LHTD took longer than 7 seconds, indicating they have poor lower-limb mobility. Additionally, all participants were categorized as having a UFOV RI of either very low or low. On the TMTA, all participants received a score below the cut-point (90 seconds), indicating their visual processing and attention were intact. Similarly, all LHTD received a score below the cut-point (180 seconds) for the TMTB, suggesting their divided attention and executive skills were within normal limits. Additionally, only one participant failed the CDT and received a score below the cut-point of 5 (out of 7), indicating they have difficulties with their working memory and executive skills. Scores on the MoCA ranged from 17 to 30 (mean 24.2 ± 2.8), and 72.2% of LHTD were considered to have mild cognitive impairment.

**Table 2.** Clinical Test Scores

Variables	*N* = 36
UFOV (in milliseconds; ms)	
Subtest 1 (ms)	17.0 ± 0.0 (17.0–17.0)
Subtest 2 (ms)	83.0 ± 58.5 (17.0–277.0)
Subtest 3 (ms)	125.5 ± 61.9 (30.0–323.0)
Risk index	
Very low	27 (75.0%)
Low	9 (25.0%)
MoCA (score out of 30)	24.2 ± 2.8 (17.0–30.0)
Non-impaired	10 (27.8%)
Mild cognitive impairment	24 (66.7%)
Moderate cognitive impairment	2 (5.6%)
TMTA	28.7 ± 10.3 (12.7–60.4)
TMTB	66.1 ± 23.2 (25.5–138.3)
CDT (score out of 7)	6.5 ± 0.9 (4.0–7.0)
RPWT	5.9 ± 1.1 (3.5–8.8)

*Note.* ms = milliseconds; s = seconds. UFOV = Useful Field of View; MoCA = Montreal Cognitive Assessment; TMTA = Trail Making Test A; TMTB = Trail Making Test B; CDT = Clock Draw Test; RPWT = Rapid Paced Walk Test.Continuous variables are shown as mean ± *SD* (range). Categorical variables are shown as frequencies (%).

Age was significantly and positively associated with UFOV-2 (*r* = .506; *p* = .002), UFOV-3 (*r* = .557; *p* < .001), and TMTA (*r* = .348; *p* = .038) scores. Moreover, years of work experience as a LHTD was significantly and positively associated with RPWT (*r* = .460; *p* = .005) and UFOV-3 (*r* = .467; *p* = .004) scores. No significant associations were found between education and gender with any clinical test score.

### Associations Between Obesity, Neck Circumference, and Fatigue

The majority of LHTD rated their overall sleep quality (in the past 7 days) as excellent (5.5%), good (75.0%), and fair (19.5%), with no LHTD rating their sleep quality as poor. LHTD reported that, on average, they get a minimum of 6.5 ± 1.4 hours (range 4.0–10.0) and a maximum of 8.1 ± 1.4 hours (range 6.0–13.0) of sleep in a 24-hour period. When grouped by obesity-status (i.e., obese vs. non-obese using BMI), obese LHTD spent significantly fewer hours sleeping when estimating their minimum (*p* = .039) and maximum (*p* = .044) number of hours of sleep in a 24-hour period (see Table 3). No significant differences emerged between fatigue or sleep quality with obesity-status. Additionally, no significant associations emerged between minimum or maximum hours slept, sleep quality, or fatigue. There were no differences in maximum hours of sleep, sleep quality, or fatigue in those with and without upper body adiposity (based on neck circumference).

**Table 3.** Fatigue Differences Between Obese and Non-Obese LHTD

Variable	Non-Obese (BMI < 30.0) (*n* = 13)	Obese (BMI ≥ 30) (*n* = 23)	Sig
Fatigue			
The amount (on a scale of 0 [not at all] to 10 [very much]) fatigue interferes with work	1.9 ± 2.0 (0.0–6.0)	2.7 ± 2.5 (0.0–8.0)	*p* = .448
The amount (on a scale of 0 [not at all] to 10 [very much]) fatigue interferes with ability to concentrate	1.9 ± 2.0 (0.0–5.0)	2.3 ± 2.4 (0.0–8.0)	*p* = .735
The amount (on a scale of 0 [not at all] to 10 [very much]) fatigue interferes with relationships	1.4 ± 2.6 (0.0–8.0)	1.5 ± 2.1 (0.0–8.0)	*p* = .641
Sleep			
Sleep quality (in past 7 days)			*p* = .605
Poor	—	—	
Fair	2 (15.4%)	5 (21.7%)	
Good	10 (77.0%)	17 (73.9%)	
Excellent	1 (7.7%)	1 (4.3%)	
On average, the minimum number of hours of sleep per 24-hour period	7.1 ± 1.2 (5.0–9.0)	6.2 ± 1.4 (4.0–10.0)	***p*** = .039*
On average, the maximum number of hours of sleep per 24-hour period	8.6 ± 1.3 (7.0–12.0)	7.9 ± 1.4 (6.0–13.0)	***p*** = .044*
Neck circumference (cm)	39.1 ± 4.5 (29.0–48.0)	42.6 ± 3.9 (36.0–51.0)	***p*** = .022*
≥43 cm	2 (15.4%)	11 (47.8%)	*p* = .075
<43 cm	11 (84.6%)	12 (52.2%)	

*Note.* Presented either using mean, standard deviations, and (range) or frequencies (and percent). B﻿oldfaced values/texts indicates statistical significance.

* *p* < .05.

### Associations Between Obesity and Workplace Productivity

Only one LHTD reported being absent from work due to injury; however, LHTD reported going to work with an injury on an average of 2.8 ± 5.4 days (range 0–30) per month. Moreover, LHTD reported being absent 0.7 ± 1.1 days (range 0–4) per month due to illness as well as going to work with an illness 0 to 31 days per month (mean 1.9 ± 5.4 days). When LHTD were grouped by obesity-status (i.e., obese vs. non-obese), obese LHTD worked significantly less (*p* = .005) days per week compared to non-obese LHTD (see Table 4). Additionally, obese LHTD took significantly more sick days (i.e., absenteeism; *p* = .022) and worked significantly more while sick and injured (i.e., presenteeism; *p* = .006 and *p* = .039, respectively) compared to non-obese LHTD. Obese LHTD also had significantly more crashes (*p* = .014) compared to non-obese LHTD. No significant associations emerged between age, gender, education, or work experience with work productivity (i.e., absenteeism and presenteeism). There were also no significant differences between those with and without upper body adiposity and days worked per week, number of sick days (i.e., absenteeism), number of days worked while sick or injured (i.e., presenteeism), number of crashes, or number of citations.

**Table 4.** Workplace Productivity Differences Between Obese and Non-Obese LHTD

Variable	Non-Obese (BMI < 30.0) (*n* = 13)	Obese (BMI ≥ 30) (*n* = 23)	Sig
Age (years)	44.7 ± 12.7 (22.0–65.0)	49.7 ± 12.0 (26.0–69.0)	*p* = .123
Completed education			
High school or less	7 (53.8%)	8 (34.8%)	*p* = .265
More than high school	6 (46.2%)	15 (65.2%)	
Work experience			
Years worked as a LHTD	12.8 ± 11.2 (2.0–36.0)	18.7 ± 12.4 (2.0–48.0)	*p* = .083
Daily working hours	14.3 ± 1.4 (12.0–17.0)	13.8 ± 1.7 (9.0–16.0)	*p* = .221
Days worked per week	6.5 ± 0.8 (5.0–7.0)	5.7 ± 0.8 (4.0–7.0)	***p*** = .005*
Number of driving citations received	2.8 ± 4.6 (0.0–15.0)	3.8 ± 4.8 (0.0–20.0)	*p* = .157
Been in a crash			*p* = .630
Yes	6 (46.2%)	13 (56.5%)	
No	7 (53.8%)	10 (43.5%)	
Number of crashes involved in	0.8 ± 0.4 (0.0–1.0)	1.6 ± 0.8 (1.0–3.0)	***p*** = .014*
Was found at-fault for the crash			*p* = .549
Yes	3 (50.0%)	7 (53.8%)	
No	3 (50.0%)	6 (46.2%)	
Fallen in the past 3 month			*p* = .501
Yes	2 (15.4%)	5 (21.7%)	
No	11 (84.6%)	18 (78.2%)	
Was injured (due to fall)			*p* = .524
Yes	1 (50.0%)	1 (20.0%)	
No	1 (50.0%)	4 (80.0%)	
Filed a WCB claim for an injury sustained on the job (in past 10 years)			***p*** = .033*
Yes	2 (15.4%)	13 (56.5%)	
No	11 (84.6%)	10 (43.5%)	
Worker productivity (per month)			
Days absent from work due to injury	—	0.3 ± 1.7 (0.0–8.0)	—
Days absent from work due to illness	0.2 ± 0.6 (0.0–2.0)	1.0 ± 1.2 (0.0–4.0)	***p*** = .022*
Days present at work with an injury	1.3 ± 3.0 (0.0–10.0)	3.7 ± 6.2 (0.0–30.0)	***p*** = .039*
Days present at work with an illness	0.1 ± 0.3 (0.0–1.0)	3.0 ± 6.5 (0.0–31.0)	***p*** = .006*

*Note.* LHTD = long-haul truck drivers. Presented either using mean, standard deviations, and (range) or frequencies and percent. Boldfaced values/texts indicates statistical significance.

* *p* < .05.

### Associations Between Obesity and Clinical Tests

When grouped by obesity-status (i.e., BMI ≥ 30.0 vs. BMI < 30.0), obese and non-obese participants performed similarly on UFOV subtest 1, subtest 3, and the RI; however, on subtest 2, obese LHTD performed significantly worse (*p* = .026) compared to non-obese participants (see Table 5). Obese and non-obese participants performed similarly on the MoCA (*p* = .125); however, when comparing their performance on the specific subtests, obese LHTD performed significantly worse on the visuospatial/executive function section (*p* = .001) and the attention/concentration section (*p* = .014). Obese LHTD took significantly longer to complete the RPWT compared to non-obese LHTD (*p* = .002). No significant differences emerged between obese and non-obese LHTD on the TMTA, TMTB, or the CDT. When grouped by upper adiposity-status, no significant differences emerged on UFOV subtest 1, subtest 2, subtest 3, or the RI, as well as MoCA (including specific subtests), TMTA, TMTB, CDT, and the RPWT.

**Table 5.** Comparisons of Clinical Test Scores Between Obese and Non-Obese LHTD

Variable	Non-Obese (BMI < 30.0) (*n* = 13)	Obese (BMI ≥ 30) (*n* = 23)	Sig
*Clinical test scores*			
UFOV (ms)			
Subtest 1 (ms)	17.0 ± 0.0 (17.0–17.0)	17.0 ± 0.0 (17.0–17.0)	—
Normal (0–30)	13 (100.0%)	23 (100.0%)	—
Subtest 2 (ms)	61.0 ± 41.2 (17.0–143.0)	96.8 ± 63.3 (24.0–277.0)	***p*** = .026*
Normal (0–99)	11 (84.6%)	16 (69.6%)	—
Some difficulty (100–349)	2 (15.4%)	7 (30.4%)	
Subtest 3 (ms)	109.5 ± 45.7 (30.0–207.0)	134.6 ± 68.7 (40.0–323.0)	*p* = .178
Normal (0–349)	13 (100.0%)	23 (100.0%)	—
Risk index			
Very low	11 (84.6%)	16 (69.6%)	*p* = .438
Low	2 (15.4%)	7 (30.4%)	
MoCA (score out of 30)	25.2 ± 2.6 (22.0–30.0)	23.7 ± 2.8 (17.0–29.0)	*p* = .125
Normal cognition (≥26)	5 (38.5%)	5 (21.7%)	*p* = .282
Cognitive impairment (CI) (≤19)	8 (61.5%)	18 (78.3%)	
Sections of the MoCA			
Executive (out of 5)	4.9 ± 0.3 (4.0–5.0)	3.7 ± 1.0 (2.0–5.0)	***p*** = .001*
Naming (out of 3)	2.9 ± 0.3 (2.0–3.0)	3.0 ± 0.0 (3.0–3.0)	—
Attention (out of 6)	5.7 ± 0.5 (5.0–6.0)	5.0 ± 1.1 (2.0–6.0)	***p*** = .014*
Language (out of 3)	1.9 ± 1.1 (0.0–3.0)	1.9 ± 1.0 (0.0–3.0)	*p* = .384
Abstraction (out of 2)	1.0 ± 0.8 (0.0–2.0)	1.7 ± 0.6 (0.0–2.0)	*p* = .997
Delayed recall (out of 5)	2.4 ± 2.0 (0.0–5.0)	2.3 ± 1.5 (0.0–5.0)	*p* = .556
Orientation (out of 6)	6.0 ± 0.0 (6.0–6.0)	6.0 ± 0.2 (5.0–6.0)	—
TMTA (s)	28.7 ± 8.7 (13.0–44.7)	28.6 ± 11.3 (12.7–60.4)	*p* = .598
Pass (≤90)	13 (100.0%)	23 (100.0%)	—
TMTB (s)	63.4 ± 21.7 (37.2–103.7)	67.6 ± 24.4 (25.5–138.3)	*p* = .305
Pass (≤180)	13 (100.0%)	23 (100.0%)	—
CDT (score out of 7)	6.5 ± 0.9 (5.0–7.0)	6.5 ± 0.9 (4.0–7.0)	*p* = .525
Pass (≥5)	13 (100.0%)	21 (91.3%)	—
Fail (<5)	—	2 (8.7%)	
RPWT (s)	5.2 ± 1.0 (3.5–6.6)	6.2 ± 1.0 (4.6–8.8)	***p*** = .002*
Adequate mobility (<7)	13 (100.0%)	19 (82.6%)	—
Poor mobility (≥7)	—	4 (17.4%)	

*Note.* LHTD = long-haul truck drivers; UFOV = Useful Field of View; MoCA = Montreal Cognitive Assessment; TMTA = Trail Making Test A; TMTB = Trail Making Test B; CDT = Clock Draw Test; RPWT =  Rapid Paced Walk Test. Presented either using mean, standard deviations, and (range) or frequencies (and percent). Higher scores on the UFOV, TMTA, TMTB, and RPWT indicate poorer performance; lower scores on the MoCA and CDT indicate poorer performance. B﻿oldfaced values/texts indicates statistical significance.

* *p* < .05.

When grouped by BMI and neck circumference, no differences emerged between obese and non-obese LHTD on UFOV subtest 1, 2, 3, and the RI, the MoCA, TMTA, TMTB, and CDT. However, obese LHTD performed significantly worse on the visuospatial/executive function subsection of the MoCA (*p* = .002) and the RPWT (*p* = .032) than non-obese LHTD. Additionally, a significant negative association was observed between systolic blood pressure and the attention/concentration section of the MoCA (*r* = −.361; *p* = .030). A near significant association was observed between diastolic blood pressure and the attention/concentration section of the MoCA (*r* = −.322; *p* = .055). Lastly, near significant effects between BMI and worker productivity, as well as BMI and past crash history, on UFOV 2 subtest scores (*p* = .053 and *p* = .064, respectively) were observed within the total sample.

## Discussion

More than half (63.9%) of the LHTD in our study were classified as obese with a BMI greater than 30.0, similar to prior Canadian (A. M. Crizzle et al., 2020, 2024; Shaw et al., 2023) and United States studies (Hege et al., 2016; Lemke et al., 2015). Our findings show that obesity significantly decreased worker productivity, even in the absence of cognitive impairment, consistent with prior studies (Borak, 2011; Lehnert et al., 2013; Neovius et al., 2009; Shrestha et al., 2016; van Duijvenbode et al., 2009). Obese LHTD in our study took significantly more sick days and worked significantly more days while sick and injured compared to non-obese LHTD. These findings are consistent with prior studies in the general population showing that obese workers take more sick days (Borak, 2011; Lehnert et al., 2013; Neovius et al., 2009; Sullivan et al., 2007; van Duijvenbode et al., 2009) and are less productive at work (Borak, 2011; Shrestha et al., 2016; Sullivan et al., 2007) compared to non-obese workers. One possible explanation for the higher rates of absenteeism observed in obese LHTD when compared to non-obese LHTD could be attributed to injury severity, as obese LHTD had significantly more worker compensation (WCB) claims from injuries sustained on the job compared to non-obese LHTD. These findings are consistent with prior studies in the general adult working population (Borak, 2011; Cawley et al., 2021). Additionally, we found that measures of worker productivity (i.e., absenteeism and presenteeism) were not associated with past crashes; therefore, it is suspected that different work hazards (e.g., ergonomic; physical; psychological) are responsible for the increased number of WCB claims observed in obese LHTD.

The findings suggest that the specific cognitive domains of divided attention and concentration are significantly worse in obese LHTD, similar to prior studies that reported obesity is associated with poorer attention/concentration (Mabry et al., 2022) in commercial vehicle drivers (including LHTD). Obese LHTD in our study performed significantly worse compared to non-obese LHTD on tests assessing divided attention/concentration (UFOV subtest 2; MoCA) and lower limb mobility (RPWT). On the MoCA, obese LHTD scored significantly poorer on the visuospatial/executive function and attention/concentration sections compared to non-obese LHTD. However, obese and non-obese LHTD performed similarly on processing speed (UFOV subtest 1; TMTA), selective attention (UFOV subtest 3; TMTA), divided attention (TMTB), general cognition (MoCA; CDT), and on the UFOV risk index. The simpler cognitive processes required for cognitive tests such as the UFOV-1, TMTA, and TMTB may remain intact for obese LHTD as they tend to experience more difficulties with higher order cognitive processes such as those required for the UFOV-2 (Classen et al., 2013; Rapoport et al., 2013).

The UFOV-2 requires participants to use a combination of visual and divided attention to process information whereas the other cognitive tests require participants to use their visual search, memory, task switching, and sequencing ability processes individually. Although, we would have expected obese LHTD to perform significantly worse on the UFOV-3 compared to non-obese LHTD, the UFOV-3 may not be sensitive enough to detect subtle differences in cognitive performance between obese and non-obese LHTD due to the larger range for normal completion time (0–349 ms) compared to UFOV-2 (0–99 ms). Additionally, the fact that UFOV-2 subtest scores, and not TMTB scores, had near significant effects between BMI and crash history (*p* = .064), suggests that UFOV-2 scores more accurately reflect the cognitive processes required for the safe operation of a motor vehicle, consistent with prior studies examining cognition and driving performance in older adults (Ball et al., 2006; Bédard et al., 2016; Clay et al., 2005). Taken together, these findings suggest that higher-order cognitive processes, particularly divided attention, may represent a mechanistic pathway linking obesity to crash risk in LHTD. Future research should examine whether impairments in divided attention mediate the association between obesity and crash involvement in LHTD. Moreover, longitudinal studies are needed to determine whether cardiometabolic risk factors contribute to subtle cognitive decline, including which cognitive domains are most affected and how the deficits progress, which in turn can increase occupational safety risks in LHTD.

The majority (72.2%) of LHTD in our study had mild cognitive impairment based on cut-point of less than 26 on the MoCA. Although only one other study reported MoCA scores in LHTD, they did not classify LHTD using defined cut-points (Bhattacharya et al., 2023). Our mean MoCA scores, however, were lower in the present study compared to the US study (mean MoCA score of 24 vs. 27). On other clinical tests, LHTD in the present study performed comparably on the TMTA (28.7 seconds vs. 30.3 seconds) and the UFOV-1 (17.0 ms vs. 15.0 ms), better on and TMTB (66.1 seconds vs. 82.4 seconds), but poorer on the UFOV-2 (83.0 ms vs. 15.0 ms) and UFOV-3 (125.5 ms vs. 109.0 ms) subtests, compared to the US sample. Despite LHTD in both the present and US studies being similar ages (47 years vs. 53 years), gender distribution (90% male vs. 94% male), and BMI (32 vs. 32), the findings suggest that LHTD within the present study exhibited more cognitive deficits compared to those in the US study. Fatigue may provide a possible explanation for the poorer performance observed on some cognitive tests in the present study; however, as data on fatigue was not collected in the US study, it is difficult to determine whether fatigue contributed to the differences observed between the two studies. Sleep deprivation (i.e., insufficient sleep quality and/or duration) is negatively associated with cognitive processes required for safe driving, including attention, reaction time, and executive function (Ren et al., 2023). These effects may help explain the poorer performance observed in the present sample on cognitive tests assessing attention (MoCA) and divided attention (UFOV-2). Given these findings, shorter sleep duration among obese LHTD may represent a potential pathway linking obesity with cognitive function and safety outcomes in this population.

In the present sample, obese drivers reported significantly fewer hours of sleep in a 24-hour period, consistent with findings from prior LHTD studies (A. M. Crizzle et al., 2024; Lemke, Houghtaling, et al., 2023; Sieber et al., 2014). Due to irregular schedules and long working hours, LHTD reported sleeping between 4.0 and 13.0 hours in a 24-hour period. In contrast, prior LHTD studies report that drivers typically average between 6.8 and 8.8 hours of sleep per 24-hours (A. M. Crizzle et al., 2024; Lemke, Houghtaling, et al., 2023; Sieber et al., 2014), suggesting that LHTD in the present study had inconsistent sleep patterns.

While we did not observe any significant associations between blood pressure and fatigue, hours slept, or sleep quality, prior studies show that hypertension can alter the cerebrovascular structure leading to insufficient blood perfusion, resulting in both cognitive impairment (Iadecola & Gottesman, 2019; Santisteban & Iadecola, 2018) and fatigue (Lemke, Thiese, et al., 2023), which together may explain the high levels of MCI in LHTD based on their MoCA score. For example, systolic blood pressure, but not diastolic blood pressure, was significantly negatively correlated with MoCA scores on the attention section, suggesting that hypertensive systolic blood pressure may be more strongly related to deficits in attention/concentration compared to other cognitive domains.

We found that blood pressure was objectively higher than what participants reported, consistent with prior studies (A. M. Crizzle et al., 2020; Shaw et al., 2023). In our study, only 25% reported being diagnosed with hypertension by a medical professional, although we found that 41.7% and 50.0% of participants had hypertensive systolic and diastolic blood pressures, respectively. This finding suggests that LHTD are likely unaware they have hypertension, especially if it is not accompanied by the symptoms of other chronic diseases. Moreover, of those who were diagnosed by a medical professional with hypertension, only 33.3% used medication to control their blood pressure (i.e., 66.6% of LHTD with diagnosed hypertension were not using medication to control their blood pressure). These findings emphasize the need for comprehensive occupational health strategies, such as incorporating screening assessments into annual medical examinations for LHTD that target hypertension and its comorbid conditions (e.g., obesity; diabetes; cardiovascular disease; sleep apnea). However, to be effective, interventions need to address the structural work conditions in the trucking industry, such as long work hours, poor nutrition, and limited opportunities for physical activity (McKeown & Crizzle, 2025), that contribute to hypertension and related health conditions.

In the United States, the Federal Motor Carrier Safety Administration (FMCSA) has outlined that stage 3 hypertension (i.e., a blood pressure exceeding 180/110 mmHg) medically disqualifies a LHTD from working; drivers with stage 1 or 2 hypertension (i.e., blood pressure between 140 and 180/90–110 mmHg) are not automatically disqualified, but instead require more frequent medical certification periods (U.S. Department of Transportation, 2014). Despite the FMCSA outlining fitness-to-drive standards for hypertension, there are no guidelines for hypertension in Canada. In the Canadian context, the Canadian Council for Motor Transport Administrators (CCMTA) outlines that hypertension alone does not disqualify a driver; it is only deemed a significant concern when accompanied by more serious conditions (e.g., sleep apnea; renal disease; glaucoma; Canadian Council of Motor Transport Administrators, 2021). As the present and other studies show that hypertension rates are high and potentially uncontrolled in LHTD (A. M. Crizzle et al., 2020; McKeown & Crizzle, 2025; Shaw et al., 2023), it is recommended that the CCMTA update their fitness-to-drive standards for commercial drivers, given the relationship between worker productivity and crashes in LHTD (A. M. Crizzle et al., 2022).

Obese LHTD in our study had significantly more crashes in their careers compared to non-obese LHTD, also consistent with findings from prior studies (Anderson et al., 2012; Chen et al., 2016; Ronna et al., 2016; Singer, 2025). Although no significant associations were found between medical conditions in LHTD and crashes (i.e., no medical conditions, one medical condition, or two or more medical conditions), a non-significant trend was observed that showed that crash risk increases with an increasing number of medical conditions. Being obese can negatively impact cognition (Anderson et al., 2012; Brito et al., 2023) and increase a driver’s risk of crashes (Hanowski et al., 2007; Thiese et al., 2017). Although we did not examine obesity and its related comorbidities on self-reported crashes, prior studies show a link between an increased number of crashes in obese drivers with various comorbid medical conditions (e.g., hypertension, diabetes, OSA) which cumulatively negatively impacts their cognition (Lentoor, 2022; Wang et al., 2016) and their ability to drive safely (Lemke, Thiese, et al., 2023; Thiese et al., 2017).

### Limitations

The primary limitation of our study is the small sample size, resulting in challenges addressing more complex relationships, such as the relationship between the number of comorbid medical conditions and crashes. The relatively small sample size is likely attributable to the study’s in-depth design, which required the completion of numerous clinical assessments and simulated drives compared to other LHTD studies that examined crashes and citations using surveys or secondary data, respectively. Additionally, we collected crash data that occurred over participants career, and we therefore cannot determine the specific date of crash occurrence, the health status of the driver at that time (e.g., obese vs. non-obese), or whether they had cognitive deficits at the time of the crash. However, our study participants did reflect the typical demographics of LHTD (e.g., age; gender; years of work experience; number of crashes) captured by prior research studies in Canada (A. Crizzle et al., 2018; A. M. Crizzle et al., 2020, 2024; McKeown & Crizzle, 2025).

We also experienced significant challenges recruiting and scheduling LHTD, given they are a mobile population with limited availability. The logistics of scheduling a participant was difficult and required coordinating efforts with Parking Services to secure a parking spot large enough for a semi-truck on the university campus. If participants preferred to park at a truck stop, an Uber was provided to drive participants to/from the university. Additionally, the hassle of traveling to campus to attend the session during their workday, at the end of their workday, or on their day off likely resulted in LHTD not participating at all. Additionally, LHTD participants may have experienced varying levels of fatigue as well as sleep quality and duration as they attended the study session during their day-off, during their workday, or at the end of their workday, which may have resulted in poorer performance on cognitive assessments. As the number of hours worked and the number of hours of slept prior to testing were not collected, this potential confounder could not be accounted for in the present analyses. Lastly, our findings are subjected to recall and social desirability biases as workplace productivity, diagnosed medical conditions, prescribed medications, sleep quantity, and crash history were all collected via questionnaires, which may have resulted in LHTD not disclosing their true rates of absenteeism, presenteeism, hours of sleep in a 24-hour period, medications, crashes, and diagnosed conditions.

## Implications for Occupational Health Practice

Our findings show that obesity in LHTD is significantly associated with decreased worker productivity, including higher absenteeism, presenteeism, and crash rates. Additionally, while cognitive impairments were prevalent in LHTD in our study, the impact of obesity on cognitive function was more nuanced than previously hypothesized, although obese LHTD had significantly poorer divided attention compared to non-obese LHTD. Given the present findings of the pilot study, future studies should examine whether impairments in divided attention mediate the relationship between obesity and crash involvement in LHTD.

**Applying Research to Occupational Health Practice**

This pilot study highlights that obesity impacts cognitive function and occupational productivity in long haul truck drivers (LHTD). Over 60% of participants were classified as obese, which was associated with increased absenteeism, presenteeism, workplace injury claims, and poorer divided attention, a cognitive domain critical for safe driving. Although cognitive impairments were not directly associated with crash history, obesity was associated with higher lifetime crash rates. A high prevalence of undiagnosed or uncontrolled hypertension was also observed, suggesting gaps in health monitoring. These findings emphasize the need for comprehensive occupational health strategies targeting obesity and hypertension. However, effective interventions must address the typical work conditions (e.g., lack of physical activity) of the industry which contribute to poor health outcomes. Incorporating cognitive and cardiovascular assessments into LHTD annual medical examinations would facilitate early detection, and when impairment is identified, prompt referral for further testing, supporting improvements in safety, productivity, and health outcomes.
